# Assessment of Soil Health Through Metagenomic Analysis of Bacterial Diversity in Russian Black Soil

**DOI:** 10.3390/microorganisms13040854

**Published:** 2025-04-09

**Authors:** Olesya O. Galanova, Nikita A. Mitkin, Albina A. Danilova, Vsevolod V. Pavshintsev, Denis A. Tsybizov, Alexander M. Zakharenko, Kirill S. Golokhvast, Tatiana V. Grigoryeva, Maria I. Markelova, Aleksey A. Vatlin

**Affiliations:** 1 Moscow Center for Advanced Studies, 123592 Moscow, Russia; 2 Institute of Ecology, Peoples’ Friendship University of Russia (RUDN University), 117198 Moscow, Russia; 3 Siberian Federal Research Center of Agrobiotechnology RAS, 630501 Krasnoobsk, Russia; danilova7alb@yandex.ru (A.A.D.);; 4 Advanced Engineering School “Agrobiotek”, Tomsk State University, 634050 Tomsk, Russia; 5 Institute of Fundamental Medicine and Biology, Kazan (Volga Region) Federal University, 420008 Kazan, Russia

**Keywords:** soil metagenome, Russian black soil, shotgun sequencing, agricultural productivity, no-till technology

## Abstract

Soil health is a critical determinant of agricultural productivity and environmental sustainability. Traditional assessment methods often fail to provide a comprehensive understanding of soil microbial communities and their functions. This study addresses this challenge by employing metagenomic techniques to assess the functionality of soil microbiomes in Russian black soil, renowned for its high fertility. We utilized shotgun metagenomic sequencing to analyze soil samples from Western Siberia subjected to different degrees of agro-soil disturbance. We identified functional genes involved in carbon (*accA*, *argG*, *acsA*, *mphE*, *miaB*), phosphorus (*phoB*, *ppa*, *pstB*, *pnp*, *phnJ*), and nitrogen (*queC*, *amiF*, *pyrG*, *guaA*, *guaB*, *napA*) metabolic pathways and associated with changes in microbial diversity, in general, and higher representation of certain bacterial species—*Bradyrhizobium* spp. The results demonstrated significant differences in microbial composition and functional potential between tillage treatments. No-Till technology and conventional tillage practices promoted beneficial microbial communities and enhanced soil health compared to long-term fallow soil. This work underscores the potential of metagenomic analysis in providing a comprehensive understanding of soil health, marking a significant advancement in the field.

## 1. Introduction

Soil health is a critical component of agricultural productivity, environmental sustainability, and overall ecosystem function. Healthy soils support plant growth by providing essential nutrients, water, and a stable structure for roots, while harboring beneficial microorganisms that enhance nutrient availability and protect plants from diseases. Soil health is linked to environmental sustainability through its role in carbon sequestration, water infiltration, and erosion control, which contribute to climate change mitigation and water quality protection [1,2]. Despite the importance of soil health, traditional assessment methods often fail to provide a comprehensive understanding of soil microbial communities and their functions [3].

Metagenomic analysis has emerged as a powerful tool for investigating soil health by characterizing microbial communities and their functional potential. This approach involves sequencing the collective genomes of microorganisms present in a soil sample, offering insights into the diversity, structure, and functional capabilities of the soil microbiome [4]. Traditional methods like culturing, PCR-based techniques, and Sanger sequencing are limited by biases and low throughput, often underrepresenting microbial diversity [5]. Metagenomics entails direct sequencing of total genomic DNA in a soil sample, bypassing the need to culture organisms and allowing the detection of microbial species that are not amenable to laboratory cultivation [6]. For example, many beneficial taxa (e.g., Azospirillum, *Rhizobium*, certain fungi) that are difficult to culture can still be identified in metagenomic data [7]. Additionally, metagenomics can assemble fragments of genomes from previously not characterized microorganisms, leading to the discovery of novel taxa or recovery of metagenome-assembled genomes (MAGs) [6]. Since shotgun sequencing does not rely on the amplification of a single gene target (which happens during 16S rRNA gene sequencing), it avoids primer biases that can skew the perceived community composition in amplicon studies. All DNA is sequenced according to its abundance in the sample, providing a more quantitative representation of dominant versus rare community members [8]. This is particularly important in soils, where certain groups might be over- or under-estimated by the 16S sequencing technique [9]. Metagenomics thus yields a truer picture of community structure, which is crucial when correlating microbial populations with soil health indicators or crop performance [7]. Additionally, unlike 16S rRNA gene sequencing that only profiles taxonomy, shotgun metagenomics reveals the functional genes and pathways present in the microbiome, linking community structure to ecological function in soil processes [6]. For instance, it allows for estimating the presence of functional genes involved in nutrient cycling—such as phosphorus, nitrogen, and carbon metabolism—[10] and even discovering previously unknown genes, such as those linked to antibiotic resistance or stress response mechanisms [11,12]. However, the high microbial diversity and variable evenness in soil pose challenges for metagenomic assembly, making it difficult to reconstruct microbial genomes from soil samples [13]. Consequently, metagenomes may remain fragmented, making it challenging to link specific genes (functions) to specific organisms [14].

Recent advances in metagenomic methods have allowed the characterization of microbial indicators of soil health as influenced by different types of tillage. These techniques can assess both compositional and functional changes in microbial communities, providing valuable insights into soil health [15]. Field-scale studies investigating microbial taxa from agricultural experiments are crucial for understanding the long-term effects of crop rotation and tillage on microbial indicator species [16] and to identify bioindicators of soil health by characterizing the changes caused by tillage [17]. Numerous studies have demonstrated that reduced tillage systems enhance microbial diversity and establish a distinct microbial community structure compared to conventionally plowed soils [18,19,20]. Metagenomic approaches provide a powerful tool for elucidating the beneficial effects of reduced tillage on a wide range of soil metabolic pathways, including carbon and nitrogen decomposition, carbon monoxide oxidation, nitrogen fixation, nitrate reduction, and phosphorus solubilization. Furthermore, metagenomics enables the identification of specific microbial taxa associated with these processes, such as *Bradyrhizobium*, *Mesorhizobium*, *Nitrososphaera*, *Phenylobacterium*, and *Rhizobium* [21].

Our research centers focus on evaluating soil health through metagenomic analysis of the microbiomes found in Russian black soil by examining soil samples from fields that have undergone different tillage methods: long-term fallow (for 16 years) and two types of grain–fallow crop rotation—conventional tillage and No-Till technology [22]. In the current study, soil samples were collected from the central forest–steppe of the Novosibirsk Priobye, located at the eastern edge of the Priob plateau, at the junction of the West Siberian Plain and the Kuznetsk Ala-Tau, Altai, and Sayan mountain systems (200–250 m a.s.l.). The region features a moderately warm, moderately humid climate, with a 38 °C annual temperature range, prolonged winters, stable snow cover (157–162 days), and soil freeze depths of 1.8–2 m. Leached black earths (Luvic Chernozem) are the most fertile soils of Western Siberia, crucial for grain crop cultivation. Since the 1980s, research has focused on soil-protective farming in this region [23], highlighting the role of reduced tillage in altering soil carbon dynamics [24]. Long-term permanent fallows are an informative tool for studying the problems of soil system stability under conditions of agrogenic degradation [25,26]. Previous research in this region has demonstrated that various tillage methods significantly impact soil quality, carbon and nitrogen stocks, and soil aggregates [27,28].

Renowned for its exceptional fertility [29], Russian black soil provides a distinctive setting for investigating soil microbial communities. Black soils have high organic matter content (often 10–15% in the surface), abundant nutrients, such as nitrogen and phosphorus, are neutral to slightly alkaline pH, and have high water-holding capacity [30]. Reduced tillage in black soil plots is associated with significantly higher soil microbial biomass and enzyme activity compared to conventional plowing [31] and promotes an increased abundance of bacterial groups involved in the nitrogen cycle [19], suggesting that decreased soil disturbance fosters microbial growth and metabolic activity. However, this relationship remains underexplored in the context of black soils in Russia. While individual studies report positive effects of reduced tillage over conventional plowing on microbial diversity and abundance, correlating with enhanced nutrient content and soil fertility [32,33,34], they lack a detailed analysis of changes in functional microbiome characteristics that could elucidate the underlying mechanisms driving soil quality improvement. By utilizing metagenomic sequencing with Illumina technology, we aimed to bridge the knowledge gap in characterizing the bacterial diversity of Russian black soil subjected to different tillage practices and to identify genes associated with phosphorus, nitrogen, and carbon cycling.

## 2. Materials and Methods

### 2.1. Study Area and Collection of Soil Samples

Soil samples were collected in August 2023 from medium-humus, medium-loamy black soil (Luvic Chernozem) in the forest–steppe region of the Ob area (coordinates: 54°53′13.5″ N, 82°59′36.7″ E). The size of the experimental plots occupied by wheat was 15 m × 18 m. Soil samples were collected at the 0–10 and 10–20 cm soil layers. The sample for analysis was an average sample of 5 individual ones, collected at each spatial replication of the experiment. On the long-term fallow, soil sampling was carried out according to the same scheme. Thus, 18 samples were obtained: 3 variants, 2 layers, 3 replicates. The soil samples were delivered to the laboratory, passed through a sieve with a cell diameter of 1 mm under sterile conditions, placed in sterile containers, and stored at a temperature of minus 80 degrees until analysis.

### 2.2. Analysis of Chemical Properties of Soil Samples

The total organic carbon (TOC) content was determined via the dichromate oxidation method [35]. The mortmass was separated by decanting the soil with water on a sieve with a cell diameter of 0.25 mm. The biomass was dried to an absolutely dry state. The carbon content was determined by thermal analysis on a Vario EL Cube elemental analyzer (Elementar Analysensysteme GmbH, Langenselbold, Germany) according to the manufacturer’s protocols. The amount of microbial biomass was determined using a substrate-induced respiration method [36]. N-NO_3_ was determined by the ionometric method. Extractant 1 N K_2_SO_4_ solution at a soil/solution ratio of 1:2. The determination of nitrates was carried out using ion-selective electrodes [37]. Phosphatase activity in the soil samples was determined using sodium phenolphthalein phosphate as a substrate (pH 6.5), with incubation at 30 °C for 60 min [38].

### 2.3. DNA Extraction

DNA was extracted using the Quick-DNA Fungal/Bacterial Microprep Kit/Quick-DNA Fecal/Soil Microbe Microprep kit (Zymo Research, Irvine, CA, USA) following the manufacturer’s protocols. The extracted DNA was quantified using a Qubit 4 Fluorometer (Thermo Fisher Scientific, Waltham, MA, USA) and quality was assessed by electrophoresis in 1% agarose gel. Additionally, DNA quality was assessed according to the manufacturer’s instructions from Agilent Technologies (Santa Clara, CA, USA) using the 2100 Bioanalyzer (Santa Clara, CA, USA).

### 2.4. Shotgun Sequencing and Bioinformatic Data Processing

DNA samples were used to create libraries using Illumina DNA Prep, (M) Tagmentation (100 ng). Shotgun sequencing using Illumina Novaseq technology (San Diego, CA, USA) was performed using the manufacturer’s standard techniques. Q30 value: ≥93.00% (percentage of sequenced bases with a Phred score ≥ 30. This value is based on the entire sequencing run). Read length: 2 × 101 bp. Demultiplexing of sequencing reads was performed using Illumina bcl2fastq (2.20). Adapters were trimmed using Skewer software (version 0.2.2) [39]. The quality of FASTQ files was analyzed using FastQC software (version 0.11.5-cegat). After trimming, 99.56% of the reads remained. The average reading depth was 21,031,903 reads per sample after trimming. The data were uploaded to the NCBI under BioProject: PRJNA1226397.

Performing the bacterial analysis, we use an integrated approach. We analyze the abundance of species, genes, and signature pairs. Taxonomic analysis was conducted on reads using Kraken2 (v.2.1.13) [40] and relative abundance was calculated using Bracken (v.2.9) at the ‘species’ levels [17]. We used the Kraken2 database pluspf, which include bacteria, fungi, protozoa, viruses, and archaea. Alpha diversity was conducted using Shannon index calculating to establish the level of taxa diversity within the groups. Beta diversity was calculated using Bray–Curtis distance matrix, NMDS method to visualize distance matrix, and PERMANOVA analysis to identify the taxa differences between the groups. The vegan (Available online: https://CRAN.R-project.org/package=vegan, accessed on 1 February 2025), adonis [41], and edgeR (v.4.4.2) [42] packages were used. To visualize the results of Bray–Curtis, we used NMDS graphs.

To perform a functional analysis, it was necessary to collect the catalog of target amino acid sequences. To do this, we compiled a list of target carbon, nitrogen, and phosphorus exchange genes and taxa that we obtained at the stage of taxonomic analysis. Using these data, we conducted multiple searches in the UniProt (v.2024-04) [43] and KEGG (v.112.0) [44] databases. The catalog was compiled from the detected sequences. All sequences satisfy the following requirements: (i) the amino acid sequence belongs to the carbon, phosphorus, or nitrogen metabolic pathways; (ii) the amino acid sequence belongs to an organism that was obtained in the results of the taxonomic analysis (up to class level); and (iii) the sequence must have at least 2 annotations in the UniProt database. Full catalog information can be viewed in Table S1. Target genes from the compiled catalog were identified within the sequencing reads using blastx Diamond (v.2.1.11) [45]. To interpret the results, we conducted the following requirements: (i) the alignment must have at least 90% homology; and (ii) the alignment length must correspond to the length of the read. The readcounts were calculated for the detected coding sequences of the genes. The calculation was performed using our own code. Then, the readcounts were normalized using the Trimmed Mean of M-values (TMM) normalization method. The results of the taxonomic and functional analysis were compared into a signature matrix consisting of pairs (taxon; gene). Matrix data were processed with TMM normalization.

### 2.5. Statistics

Statistical analysis of the chemical parameters of the soil samples was performed applying one-way ANOVA with post hoc Tukey’s test. The data are presented as the mean ± standard error of the mean. Differences between the groups were considered to be significant at *p* < 0.05. Statistical analysis was performed using Prism 9.1 (GraphPad, San Diego, CA, USA).

The Wilcoxon–Mann–Whitney test (U-criterion) was used to identify statistically significant differences in the abundance of taxa, genes, and signature pairs between the soil tillage type groups. After that, we used False Positive Rate correction (FDR) by the Benjamini–Hochberg method based on U-criterion *p*-values. Further in the work, differences in the relative abundance of taxa, genes, or signature pairs for which the FDR *p*-values are less than 0.05 will be called ‘Statistically significant’.

To visualize the results, we selected taxa, genes, and signature pairs with statistically significant differences in abundance, calculated the median value for each soil tillage type group, and built graphs. The median values are chosen as the most convenient for presenting the result by group. The stats, matplotlib, numpy, and pandas libraries were used (available online: https://matplotlib.org/stable/index.html, (accessed on 1 February 2025), https://numpy.org/doc/, (accessed on 1 February 2025), https://api.semanticscholar.org/CorpusID:61539023, (accessed on 1 February 2025)).

## 3. Results

### 3.1. Assessment of Soil Chemical Parameters

The chemical properties of the soil under different tillage systems are summarized in Table 1. Total organic carbon (TOC) did not differ significantly among treatments (*p* = 0.38). No-till technology resulted in significantly higher carbon content in mortmass (1133 ± 61.2 mg/kg) compared to conventional tillage (817 ± 51.4 mg/kg). Both tillage practices led to markedly increased carbon content in mortmass compared to long-term fallow soil (67 ± 12.5 mg/kg). Microbial biomass carbon was significantly higher in No-Till (120 ± 12.5 μg/g) and conventional tillage (100 ± 10.2 μg/g) than in the fallow samples (30 ± 5.5 μg/g), though no statistically significant differences were observed between the two tillage systems. Nitrate content (N-NO₃) exhibited an inverse trend, with the highest concentration in long-term fallow (78.6 ± 13.95 mg/kg), followed by conventional tillage (19.8 ± 7.78 mg/kg), and the lowest in No-Till (4.4 ± 0.22 mg/kg). Phosphatase activity was markedly higher in No-Till soils (26.2 ± 0.84 μg P_2_O_5_/g/h) compared to conventional tillage (14.6 ± 0.64 μg P_2_O_5_/g/h) and fallow (13.8 ± 0.43 μg P_2_O_5_/g/h), with no statistically significant distinction detected between conventional tillage and fallow.

### 3.2. Taxonomy Abundance and Diversity Analysis

**Figure 1:**
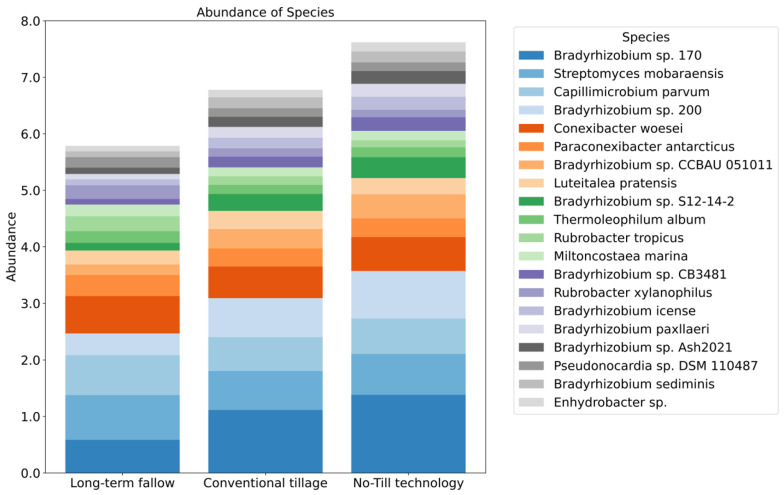
Comparison of median values of relative abundance of top-20 species for groups of leached black earths. Relative abundance of species in % is located on Y-axis. On X-axis, there are subgroups of soil samples.

We identified 428 species with statistically significant differences in relative abundance between all the groups of soil tillage. The graph (Figure 1) shows the top-20 of these species with median values for each group. The relative abundance of species *Streptomyces mobaraensis*, *Capillimicrobium parvum*, *Conexibacter woesei*, *Bradyrhizobium* sp. 170, *Bradyrhizobium* sp. 200, *Bradyrhizobium* sp. CCBAU 051011, and *Bradyrhizobium* sp. S12-14-2 differs the most between the groups of tillage types. The differences in the abundance of these species are more than 0.5%. The abundance of species *Streptomyces mobaraensis*, *Capillimicrobium parvum*, and *Conexibacter woesei* is higher for soil samples with long-term fallow in comparison with the conventional tillage and No-Till technology groups. The species *Bradyrhizobium* sp. 170 is the most represented in the subgroup of No-Till technology and the least represented in the long-term fallow group, as well as the species *Bradyrhizobium* sp. 200, *Bradyrhizobium* sp. CCBAU 051011, *Bradyrhizobium* sp. S12-14-2, and *Bradyrhizobium license*.

**Figure 2:**
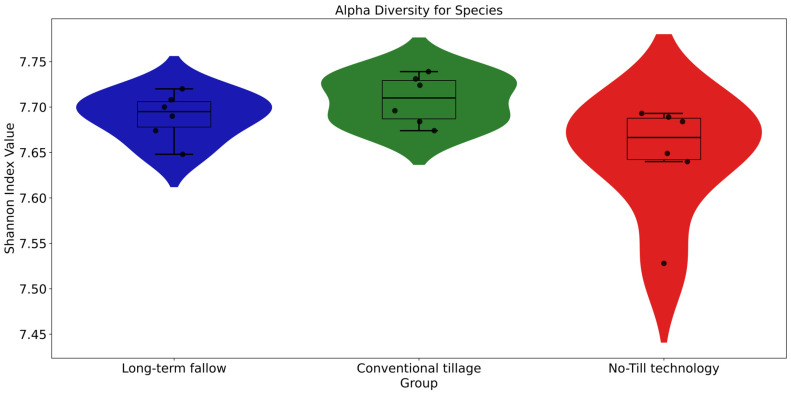
The alpha diversity of the taxonomic composition of the leached black earths. The values of the Shannon index are located on the Y-axis. The X-axis shows the subgroups of the soil samples. The shape of the graph represents the distribution density of the Shannon index. The boxplot shows the median, quartiles, and spread of the Shannon index among the data.

The alpha diversity analysis revealed a high level of species diversity, with no statistically significant differences observed among the subgroups subjected to varying tillage practices. The Shannon diversity index, averaging approximately 7.7 across all groups, indicates a notably high level of species richness and evenness (Figure 2).

**Figure 3:**
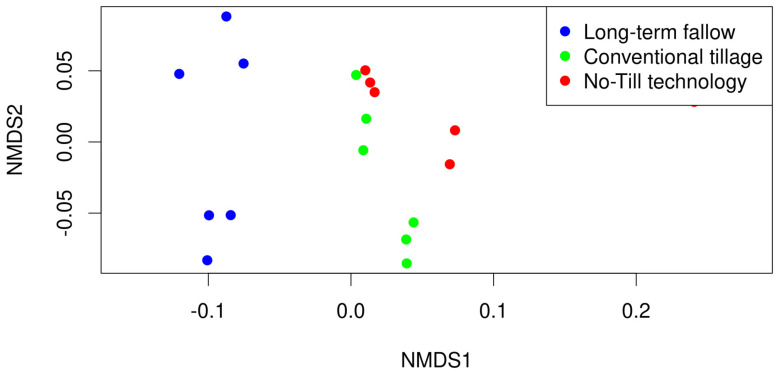
The beta diversity of the taxonomic composition of the leached black earths at the level of species. The NMDS graph shows the clustering of soil samples (dots on the figure). The blue dots represent soil samples from the sample subgroups with long-term fallow, the green dots are for the soil samples with conventional tillage, and the red dots are for the soil samples with No-Till technology.

The beta diversity analysis, conducted using the Bray–Curtis dissimilarity matrix and PERMANOVA, revealed statistically significant differences among the subgroups. The NMDS graph (Figure 3) further corroborates these findings, demonstrating clear clustering of samples into three distinct subgroups corresponding to the tillage practices; the results of the PERMANOVA test indicate that this result is statistically significant (*p*-value = 0.01). The stress-value for NMDS analysis was 0.05. At the same time, the data exhibited considerable dispersion at the species level. The PERMANOVA results indicated that approximately 40% of the observed variability in the data set can be attributed to the classification of soil samples into the three tillage practice groups.

### 3.3. Functional Analysis

**Figure 4:**
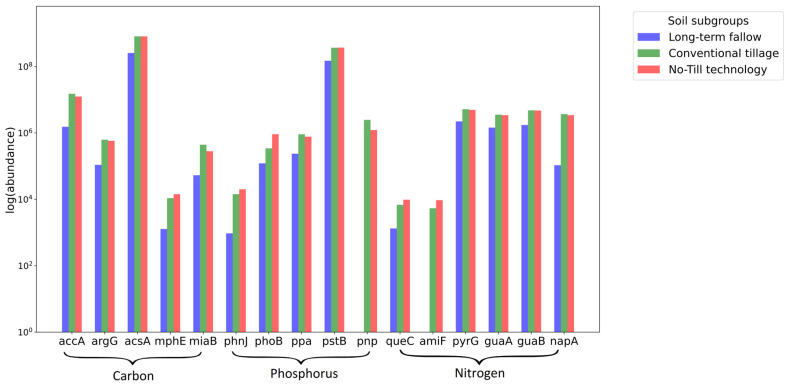
A comparison of the median values of the relative representation of genes for the groups of leached black earths. The logarithm of relative abundance is located on the Y-axis. Gene names with statistically significant differences in relative abundance between the soil groups are located on the X-axis. The blue bars represent abundance for the long-term fallow group, the green bars represent the abundance for the conventional tillage group, and the red bars represent the abundance for the No-Till technology group.

The genes of carbon, nitrogen, and phosphorus metabolism were searched using the catalog of homologues and reads. The genes with statistically significant differences in relative abundance between the soil subgroups are presented in Figure 4.

The relative abundance of the genes from Figure 4 is presented as the median relative abundance of the gene in the soil sample subgroups (Appendix A, Table A1). The annotation (function and belonging to the metabolic pathway) for these genes is performed in Table 2.

The relative abundance of the genes differs significantly for soil subgroup ‘Long-term fallow’ and ‘Conventional tillage’, ‘Long-term fallow’ and ‘No-Till technology’. The difference between ‘Conventional tillage’ and ‘No-Till technology’ is not statistically significant. In all cases, the relative abundance of genes for ‘Long-term fallow’ is significantly lower than for the other subgroups. Genes *acsA*, *accA*, and *pstB* are the most represented (much more than 10^6^). Genes *amiF* and *pnp* are not represented in ‘Long-term fallow’ (the median value of the readcounts is zero). Genes *mphE*, *phnJ*, and *queC* are presented in ‘Long-term fallow’ at a significantly lower level compared to the other subgroups and genes (lower than 10^4^).

### 3.4. Signature Analysis

**Figure 5:**
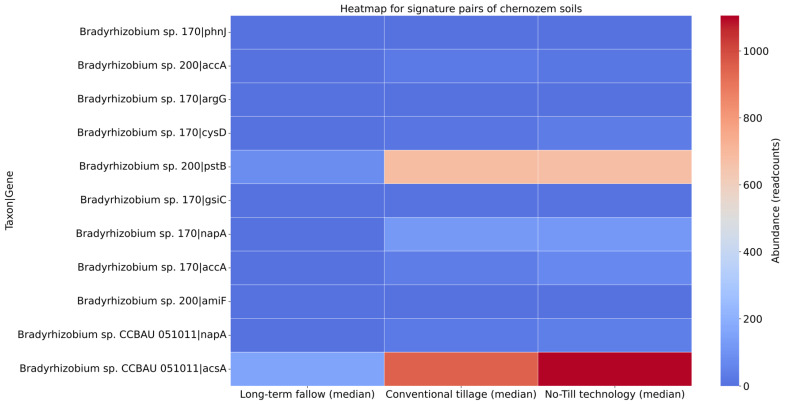
The heatmap for signature pairs, compiled according to their median relative abundance. The center of the scale is set at 500 readcounts. ‘*Bradyrhizobium* sp. CCBAU 051011|acsA’ and ‘*Bradyrhizobium* sp. 200|pstB’ represent the highest abundance.

To provide the results, all pairs containing the species with the largest difference in representation between the subgroups were selected. These species are *Streptomyces mobaraensis*, *Capillimicrobium parvum*, *Conexibacter woesei*, *Bradyrhizobium* sp. 170, *Bradyrhizobium* sp. 200, *Bradyrhizobium* sp. CCBAU 051011, and *Bradyrhizobium* sp. S12-14-2. Then, a heatmap was built to visualize the differences in their median relative abundance (Figure 5). Here, we provide only the signature pairs with statistically significant differences in abundance.

The heatmap shows that the signature pair ‘*Bradyrhizobium* sp. CCBAU 051011|acsA’ has the highest abundance for ‘No-Till technology’ (over 1000 readcounts in median) and ‘Conventional tillage’ (about 1000 readcounts in median) in comparison with ‘Long-term fallow’. The signature pair ‘*Bradyrhizobium* sp. 200|pstB’ has a much high abundance (about 700 readcounts) in the tilled groups compared with the ‘Long-term fallow’ subgroup. These results support the results of taxonomic and functional analysis.

## 4. Discussion

Modern agriculture faces several challenges such as soil degradation, loss of fertility, and the need for sustainable agricultural practices. Integrated soil health assessment methods, particularly through metagenomic analysis, can help to address these challenges. Our study contributes to this growing body of knowledge by employing metagenomic tools to evaluate the microbial communities and functional potential of Russian black soil (chernozem), a soil type renowned for its high fertility and agricultural importance [29]. Despite its inherent fertility, chernozem is not immune to degradation, and its sustainable management is critical to preserving its productivity [46]. By employing metagenomic techniques, we were able to provide a comprehensive understanding of the microbial communities inhabiting this soil type and their functional potential. Using advanced metagenomic methods, we have gained insight into microbial diversity and genes that may be critical for maintaining soil health and productivity.

Our key findings highlight significant differences in microbial community composition under different tillage regimes. No-Till technology and conventional tillage practices promoted both bacterial diversity, according to the relative abundance of specific taxa in the groups (Figure 1) and alpha diversity (Figure 2) and beta diversity analysis (Figure 3), which may contribute to improved soil health through enhanced nutrient cycling and organic matter decomposition compared to long-term bare fallow soil. The relative representation of the major soil species, Streptomyces mobaraensis, Capillimicrobium parvum and Conexibacter woesei, inversely correlates with the increase in total microbial diversity (Figure 1). S. mobaraensis and C. woesei are known to form extended hyphal networks, and their abundance is associated with stable, structured soils where they thrive in protective microhabitats within intact soil aggregates [47,48]. C. parvum, a small-sized actinobacterium, is likely sensitive to rapid changes in soil moisture and organic matter distribution, which occur while applying regular tillage procedures [49]. Both tillage practices that break up soil aggregates can expose the described major bacterial species to harsher conditions—reducing their capacity to colonize and persist in the soil matrix, but at the same time, enabling the growth and development of other bacterial species. According to the alpha diversity results, we can see high levels of species richness and evenness [50], which suggests a substantial heterogeneity in species composition within each soil subgroup. These findings correspond to the increase in total carbon in microbial biomass (Table 1) and are consistent with previous studies demonstrating the positive impact of permanent tillage on soil microbial diversity and community stability [51]. At the same time, No-Till technology, which is considered a reduced tillage practice [52], was associated with a more heterogeneous soil environment than in the case of conventional tillage, providing diverse ecological niches that support a wider range of microbial lifestyles. This diversity is crucial for maintaining the resilience of soil ecosystems and their ability to provide essential services, such as nutrient availability and disease suppression, to plants and their environment [53]. The increased microbial diversity under reduced tillage conditions is consistent with previous findings [18,19,20] and can be attributed to the maintenance of soil structure and organic matter, which contribute to a more stable and supportive habitat for microbial communities. Additionally, these patterns align with observations in other fertile black soil systems. For example, in Northeast China’s Mollisol (black soil) region, no-tillage combined with residue mulching significantly increased the alpha diversity of soil bacteria and altered community composition, while increasing soil organic carbon and nutrient levels [54,55]. This parallel suggests that the benefits of reduced tillage on microbial diversity and function observed in our study are not unique to this Russian black soil but are likely applicable to other black soil-rich agricultural regions under comparable management practices. In contrast, conventional tillage disrupts soil aggregates, leading to the loss of organic matter, which results in less extensive microbial diversity [56]. In general, both tillage methods positively influenced soil microbial diversity, with reduced tillage No-Till technology demonstrating greater effectiveness.

The results of the study indicate that both treatment technologies led to a significant increase in the abundance of a certain microbial taxa—*Bradyrhizobium* spp.—which is in agreement with previously published data [57]. Bradyrhizobium spp. are well known symbiotic nitrogen-fixing bacteria that form nodules on the roots of leguminous plants [58] and are responsible for heavy metal resistance and bioremediation in a long-term heavy metal-contaminated ecosystem [59]. *Bradyrhizobium* spp. have been shown to stimulate plant growth and improve plant resistance to biotic and abiotic stresses, further highlighting their importance as indicators of soil health and plant vitality [60,61]. The extended presence of Bradyrhizobium spp. under reduced tillage conditions (No-Till technology) suggests that this practice not only enhances soil fertility but also contributes to the resilience of soil ecosystems in the face of environmental stressors.

The functional analysis (Figure 4) revealed that genes associated with carbon metabolism, such as *accA*, *argG*, *acsA*, *mphE*, and *miaB*, were more abundant in soils subjected to No-Till technology and conventional tillage practices. Among them, *accA* (acetyl-coenzyme A carboxylase) and *acsA* (acetyl-coenzyme A synthetase) are especially crucial for carbon fixation and the synthesis of acetyl-CoA, a key intermediate in the carbon cycle [62]. The increased abundance of these genes due to the application of tillage suggests enhanced carbon sequestration and organic matter decomposition, which are critical for maintaining soil fertility [56]. This assumption is supported by the fact that both methods of soil treatment led to significantly higher carbon content in microbial biomass and the mortmass, as well as increased CO_2_ emission, compared to untreated soil (Table 1). Signature analysis (Figure 5) identified *Bradyrhizobium* sp. CCBAU 051011 as a crucial bacterial species associated with the increased abundance of *acsA* in tilled soils, highlighting its significant role in carbon fixation processes.

The optimization of carbon and nitrogen metabolism in soils under agricultural practices has been extensively studied in the literature. However, significantly less attention has been given to the dynamics of phosphorus metabolism [63]. In particular, the influence of soil cultivation methods on phosphorus metabolism remains underexplored. Nonetheless, a global meta-analysis of 5876 observations demonstrated that soil conservation practices significantly enhance soil phosphatase activity [64]. Our findings provide additional support for this conclusion through the lens of soil metagenomic analysis. Phosphorus metabolism was significantly affected by tillage practices, with genes such as *phoB*, *ppa*, *pstB*, *pnp*, and *phnJ* exhibiting higher abundance in soils subjected to treatment. These genes play essential roles in phosphorus uptake and regulation, processes vital for sustaining soil phosphorus availability and supporting plant nutrition [65]. Specifically, *phoB*, *ppa*, *phnJ*, and *pstB* encode key proteins involved in solubilizing soil phosphate, thereby enhancing its accessibility to plants [10]. For instance, *phoB* is crucial for the regulation of phosphate uptake and solubilization as well as P-starvation response, since it controls the expression of phosphatases and phosphate transporters [66]. In tilled soils, the higher abundance of *phoB* suggests an adaptive response to phosphorus scarcity, promoting the solubilization of inorganic phosphate and enhancing its availability to plants. The increase in phoB abundance was 3 times for plowing and 7 times for No-Till compared to fallow, which may be a sign of optimization of phosphorus metabolism conditions under soil conservation technology. The higher presence of *ppa*, encoding an inorganic pyrophosphatase, corresponds to elevated phosphatase activity (Table 1) in tilled soils, which is potentially associated with enhanced mineralization of organic phosphorus compounds, releasing orthophosphate for plant uptake [67]. The abundance of *phnJ*, a gene associated with the mineralization of organic phosphorus compounds [68], increased by two orders of magnitude compared to fallow conditions. The abundance of the *pstB* gene, encoding a phosphate transporter protein involved in phosphorus uptake and transport, was 2.5 times higher in tilled soils than fallow. Signature analysis (Figure 5) further highlighted that the elevated presence of *pstB* in tilled soils is linked to the increased abundance of *Bradyrhizobium* sp. 200, suggesting that this species plays a significant role in phosphorus solubilization.

In the context of nitrogen metabolism, genes such as *queC*, *amiF*, *pyrG*, *guaA*, *guaB*, and *napA* were more abundant in soils subjected to tillage practices. These genes are essential for nitrogen fixation and nitrate reduction, processes that are critical for soil fertility and plant growth. The increased abundance of these genes under tillage conditions is consistent with the higher levels of microbial biomass and the significant reduction in nitrate–nitrogen (N-NO_3_) soil content (Table 1). N-NO_3_ reduction is likely due to the enhanced activity of microbial communities carrying nitrogen metabolism genes, which promotes the immobilization of nitrogen in organic forms and reduces nitrate leaching [69]. Nitrate leaching is a major environmental concern, as it can lead to the contamination of groundwater and surface water bodies, contributing to eutrophication and other ecological imbalances [70]. By minimizing nitrate losses, tillage practices not only improve soil health but also contribute to broader environmental sustainability. The high presence of nitrogen metabolism genes in tilled soils is in agreement with the increased abundance of *Bradyrhizobium* spp., renowned for their ability to fix free nitrogen through symbiotic relationships with legumes and reported to carry the genes of this cluster [71]. However, none of the classical nif (nitrogen fixation) genes were detected in our metagenomic data. This apparent absence of nif gene sequences could indicate that the *Bradyrhizobium* populations present are predominantly free-living strains lacking the symbiotic nitrogen-fixation island, as has been observed in some non-diazotrophic *Bradyrhizobium* from other soils [61]. Environmental conditions might also play a role; for example, the absence of legume hosts and a relatively sufficient soil nitrogen status may reduce the need to retain nif genes, leaving them below the detection threshold of our sequencing.

Overall, our study reinforces the role of microbiome-driven processes in sustainable soil management. By showing that reduced tillage practices (like No-Till) enhance beneficial microbial communities and nutrient cycling in black soil, we contribute to the broader global effort to promote sustainable agriculture. These results echo calls to harness soil biodiversity for improving fertility and resilience [72], and they demonstrate how managing the soil microbiome can help balance agricultural productivity with environmental health on a long-term basis.

## 5. Conclusions

This study demonstrates that different tillage practices significantly influence the functional genes and microbial taxa associated with key biogeochemical cycles in Russian black soil. No-tillage technology and conventional tillage practices promote beneficial microbial communities and enhance soil health by improving nutrient cycling and organic matter decomposition. The increased abundance of genes involved in carbon, nitrogen, and phosphorus metabolism, as well as the microbial taxa responsible for these processes, underscores the importance of adopting sustainable tillage practices to maintain soil fertility and productivity. The use of metagenomic analysis provides a comprehensive understanding of soil microbial communities and their functions, demonstrating high correlation with traditional widely used physical and chemical methods evaluating soil quality/fertility and offering valuable insights for the development of sustainable agricultural practices.

## Figures and Tables

**Table 1:** Properties of leached medium-humus, medium-loamy chernozem (Luvic Chernozem) of Ob forest–steppe subjected to different types of tillage. Mean ± Std Err.

Soil Indicator	Long-Term Fallow	Conventional Tillage	No-Till Technology
TOC %	4.1 ± 0.07	4.2 ± 0.06	4.2 ± 0.04
C in mortmass, mg/kg	67 ± 12.5	817 ± 51.4	1133 ± 61.2
C in microbial biomass, μg/g	30 ± 5.5	100 ± 10,2	120 ± 12.5
N-NO_3_, mg/kg	78.6 ± 13.95	19.8 ± 7,78	4.4 ± 0.22
Phosphatase activity, μg, P_2_O_5_/g per hour	13.8 ± 0.43	14.6 ± 0,64	26.2 ± 0.84

**Table 2:** Annotation for genes with statistically significant differences in relative abundance for their function and which metabolic pathway they belong to (carbon, nitrogen, phosphorus).

Gene Name	Enzyme Name	KEGG Enzyme Entry	Metabolic Pathway
*accA*	Acetyl-coenzyme A carboxylase carboxyl transferase subunit alpha	2.1.3.15	carbon
*argG*	Argininosuccinate synthase (Forming carbon-nitrogen bonds)	6.3.4.5	
*acsA*	Acetyl-coenzyme A synthetase	6.2.1.1	
*mphE*	4-hydroxy-2-oxovalerate aldolase	4.1.3.39	
*miaB*	tRNA-2-methylthio-N(6)-dimethylallyladenosine synthase (catalyzes methylation)	2.8.4.3	
*phnJ*	Alpha-D-ribose 1-methylphosphonate 5-phosphate C-P lyase	4.7.1.1	phosphorus
*phoB*	Phosphate regulon transcriptional regulatory protein	3.6.1.11	
*ppa*	Inorganic pyrophosphatase	3.6.1.1	
*pstB*	Phosphate import ATP-binding protein	7.3.2.1	
*pnp*	Polyribonucleotide nucleotidyltransferase (catalyzes the phosphorolysis)	2.7.7.8	
*queC*	7-cyano-7-deazaguanine synthase (Forming carbon-nitrogen bonds)	6.3.4.20	nitrogen
*amiF*	Formamidase (Acting on carbon-nitrogen bonds)	3.5.1.49	
*pyrG*	CTP synthase (glutamine hydrolysing) (Forming carbon-nitrogen bonds)	6.3.4.2	
*guaA*	GMP synthase [glutamine-hydrolyzing] (Forming carbon-nitrogen bonds)	6.3.5.2	
*guaB*	Inosine-5′-monophosphate dehydrogenase (Acting on the CH-OH group of donors)	1.1.1.205	
*napA*	Periplasmic nitrate reductase	1.9.6.1	

## Data Availability

The original contributions presented in this study are included in the article/Supplementary Material. Further inquiries can be directed to the corresponding author.

## Supplementary Materials

The following supporting information can be downloaded at: https://www.mdpi.com/article/10.3390/microorganisms13040854/s1, Table S1: Catalog information.

## Appendix A

**Table A1:** Median relative abundances for genes with statistically significant differences.

Gene	Long-Term Fallow	Conventional Tillage (Median)	No-Till Technology (Median)
*accA*	1.53 × 10^6^	15.15 × 10^6^	12.48 × 10^6^
*acsA*	2.55 × 10^8^	8.09 × 10^8^	8.08 × 10^8^
*amiF*	0	5.38 × 10^3^	9.43 × 10^3^
*argG*	108.81 × 10^3^	624.34 × 10^3^	578.59 × 10^3^
*guaA*	1.44 × 10^6^	3.54 × 10^6^	3.41 × 10^6^
*guaB*	1.73 × 10^6^	4.79 × 10^6^	4.72 × 10^6^
*miaB*	53.03 × 10^3^	441.45 × 10^3^	281.52 × 10^3^
*mphE*	1.27 × 10^3^	10.87 × 10^3^	14.29 × 10^3^
*napA*	106.36 × 10^3^	3.67 × 10^6^	3.42 × 10^6^
*phnJ*	0.94 × 10^3^	14.27 × 10^3^	20.07 × 10^3^
*phoB*	121.40 × 10^3^	343.04 × 10^3^	913.79 × 10^3^
*pnp*	0	2.46 × 10^6^	1.22 × 10^6^
*ppa*	236.70 × 10^3^	910.72 × 10^3^	772.52 × 10^3^
*pstB*	1.49 × 10^8^	3.68 × 10^8^	3.72 × 10^8^
*pyrG*	2.22 × 10^6^	5.18 × 10^6^	4.96 × 10^6^
*queC*	1.32 × 10^3^	6.84 × 10^3^	9.72 × 10^3^

## References

[1] Wilhelm R.C., Amsili J.P., Kurtz K.S.M., van Es H.M., Buckley D.H. (2023). Ecological Insights into Soil Health According to the Genomic Traits and Environment-Wide Associations of Bacteria in Agricultural Soils. ISME Commun..

[2] Zapata J.D.D., Florez J.E.M., Alvarez D.L. (2023). Metagenomics Approaches to Understanding Soil Health in Environmental Research—A Review. Soil Sci. Ann..

[3] Chang T., Feng G., Paul V., Adeli A., Brooks J. (2022). Soil Health Assessment Methods: Progress, Applications and Comparison. Advances in Agronomy.

[4] Prosser J.I. (2015). Dispersing Misconceptions and Identifying Opportunities for the Use of “omics” in Soil Microbial Ecology. Nat. Rev. Microbiol..

[5] Caporaso J.G., Lauber C.L., Walters W.A., Berg-Lyons D., Huntley J., Fierer N., Owens S.M., Betley J., Fraser L., Bauer M. (2012). Ultra-High-Throughput Microbial Community Analysis on the Illumina HiSeq and MiSeq Platforms. ISME J..

[6] Garg D., Patel N., Rawat A., Rosado A.S. (2024). Cutting Edge Tools in the Field of Soil Microbiology. Curr. Res. Microb. Sci..

[7] Omotayo O.P., Igiehon O.N., Babalola O.O. (2022). Microbial Genes of Agricultural Importance in Maize Rhizosphere Unveiled Through Shotgun Metagenomics. Span. J. Soil Sci..

[8] Becker B., Pushkareva E. (2023). Metagenomics Provides a Deeper Assessment of the Diversity of Bacterial Communities in Polar Soils Than Metabarcoding. Genes.

[9] Poretsky R., Rodriguez-R L.M., Luo C., Tsementzi D., Konstantinidis K.T. (2014). Strengths and Limitations of 16S rRNA Gene Amplicon Sequencing in Revealing Temporal Microbial Community Dynamics. PLoS ONE.

[10] Wu X., Rensing C., Han D., Xiao K.-Q., Dai Y., Tang Z., Liesack W., Peng J., Cui Z., Zhang F. (2022). Genome-Resolved Metagenomics Reveals Distinct Phosphorus Acquisition Strategies between Soil Microbiomes. mSystems.

[11] Yuan K., Yu K., Yang R., Zhang Q., Yang Y., Chen E., Lin L., Luan T., Chen W., Chen B. (2019). Metagenomic Characterization of Antibiotic Resistance Genes in Antarctic Soils. Ecotoxicol. Environ. Saf..

[12] Xing C., Chen J., Zheng X., Chen L., Chen M., Wang L., Li X. (2020). Functional Metagenomic Exploration Identifies Novel Prokaryotic Copper Resistance Genes from the Soil Microbiome. Metallomics.

[13] White R.A., Bottos E.M., Roy Chowdhury T., Zucker J.D., Brislawn C.J., Nicora C.D., Fansler S.J., Glaesemann K.R., Glass K., Jansson J.K. (2016). Moleculo Long-Read Sequencing Facilitates Assembly and Genomic Binning from Complex Soil Metagenomes. mSystems.

[14] Anthony W.E., Allison S.D., Broderick C.M., Chavez Rodriguez L., Clum A., Cross H., Eloe-Fadrosh E., Evans S., Fairbanks D., Gallery R. (2024). From Soil to Sequence: Filling the Critical Gap in Genome-Resolved Metagenomics Is Essential to the Future of Soil Microbial Ecology. Environ. Microbiome.

[15] Song W., Wang Y., Peng B., Yang L., Gao J., Xiao C. (2023). Structure and Function of Microbiomes in the Rhizosphere and Endosphere Response to Temperature and Precipitation Variation in Inner Mongolia Steppes. Front. Plant Sci..

[16] Behnke G.D., Kim N., Zabaloy M.C., Riggins C.W., Rodriguez-Zas S., Villamil M.B. (2021). Soil Microbial Indicators within Rotations and Tillage Systems. Microorganisms.

[17] Kim N., Zabaloy M.C., Riggins C.W., Rodríguez-Zas S., Villamil M.B. (2020). Microbial Shifts Following Five Years of Cover Cropping and Tillage Practices in Fertile Agroecosystems. Microorganisms.

[18] Srour A.Y., Ammar H.A., Subedi A., Pimentel M., Cook R.L., Bond J., Fakhoury A.M. (2020). Microbial Communities Associated with Long-Term Tillage and Fertility Treatments in a Corn-Soybean Cropping System. Front. Microbiol..

[19] Domnariu H., Trippe K.M., Botez F., Partal E., Postolache C. (2025). Long-Term Impact of Tillage on Microbial Communities of an Eastern European Chernozem. Sci. Rep..

[20] Sipilä T.P., Yrjälä K., Alakukku L., Palojärvi A. (2012). Cross-Site Soil Microbial Communities under Tillage Regimes: Fungistasis and Microbial Biomarkers. Appl. Environ. Microbiol..

[21] Hu X., Liu J., Liang A., Gu H., Liu Z., Jin J., Wang G. (2025). Soil Metagenomics Reveals Reduced Tillage Improves Soil Functional Profiles of Carbon, Nitrogen, and Phosphorus Cycling in Bulk and Rhizosphere Soils. Agric. Ecosyst. Environ..

[22] Coughenour C.M. (2009). Innovating Conservation Agriculture: The Case of No-Till Cropping. Rural Sociol..

[23] Kiryushin V.K., Vlasenko A.N., Kalichkin V.K. (2002). Adaptive Landscape Farming Systems of the Novosibirsk Region. Novosib. Izd. SO RASKhN Publ. House Sib. Branch Russ. Acad. Sci..

[24] Danilova A.A. (2018). Biodynamics of Arable Soil at Different Content of Organic Matter. Novosib. SFNCA RAS.

[25] Neal A.L., Bacq-Labreuil A., Zhang X., Clark I.M., Coleman K., Mooney S.J., Ritz K., Crawford J.W. (2020). Soil as an Extended Composite Phenotype of the Microbial Metagenome. Sci. Rep..

[26] Wu Y., Kemmitt S., White R.P., Xu J., Brookes P.C. (2012). Carbon Dynamics in a 60 Year Fallowed Loamy-Sand Soil Compared to That in a 60 Year Permanent Arable or Permanent Grassland UK Soil. Plant Soil.

[27] Zheng H., Liu W., Zheng J., Luo Y., Li R., Wang H., Qi H. (2018). Effect of Long-Term Tillage on Soil Aggregates and Aggregate-Associated Carbon in Black Soil of Northeast China. PLoS ONE.

[28] Wang L., Qi S., Gao W., Luo Y., Hou Y., Liang Y., Zheng H., Zhang S., Li R., Wang M. (2023). Eight-Year Tillage in Black Soil, Effects on Soil Aggregates, and Carbon and Nitrogen Stock. Sci. Rep..

[29] Sorokin A., Owens P., Lang V., Jiang Z., Micheli E., Krasilnikov P. (2021). “Black Soils” in the Russian Soil Classification System, the US Soil Taxonomy and the WRB: Quantitative Correlation and Implications for Pedodiversity Assessment. CATENA.

[30] Medinski T., Freese D., Reitz T. (2018). Changes in Soil Phosphorus Balance and Phosphorus-Use Efficiency under Long-Term Fertilization Conducted on Agriculturally Used Chernozem in Germany. Can. J. Soil Sci..

[31] Balla Kovács A., Juhász E.K., Béni Á., Kincses I., Tállai M., Sándor Z., Kátai J., Rátonyi T., Kremper R. (2024). Changes in Microbial Community and Activity of Chernozem Soil under Different Management Systems in a Long-Term Field Experiment in Hungary. Agronomy.

[32] Naumova N., Barsukov P., Baturina O., Rusalimova O., Kabilov M. (2022). Soil Mycobiome Diversity under Different Tillage Practices in the South of West Siberia. Life.

[33] Naumova N., Barsukov P., Baturina O., Rusalimova O., Kabilov M. (2023). West-Siberian Chernozem: How Vegetation and Tillage Shape Its Bacteriobiome. Microorganisms.

[34] Khmelevtsova L.E., Sazykin I.S., Azhogina T.N., Sazykina M.A. (2022). Influence of Agricultural Practices on Bacterial Community of Cultivated Soils. Agriculture.

[35] Heanes D.L. (1984). Determination of Total organic-C in Soils by an Improved Chromic Acid Digestion and Spectrophotometric Procedure. Commun. Soil Sci. Plant Anal..

[36] Anderson J.P.E., Domsch K.H. (1978). A Physiological Method for the Quantitative Measurement of Microbial Biomass in Soils. Soil Biol. Biochem..

[37] Milham P.J., Awad A.S., Paull R.E., Bull J.H. (1970). Analysis of Plants, Soils and Waters for Nitrate by Using an Ion-Selective Electrode. Analyst.

[38] Khaziev F. (1976). Enzymatic Activity of Soils. Mosc. Nauka Publ..

[39] Jiang H., Lei R., Ding S.-W., Zhu S. (2014). Skewer: A Fast and Accurate Adapter Trimmer for next-Generation Sequencing Paired-End Reads. BMC Bioinform..

[40] Wood D.E., Lu J., Langmead B. (2019). Improved Metagenomic Analysis with Kraken 2. Genome Biol..

[41] Zapala M.A., Schork N.J. (2006). Multivariate Regression Analysis of Distance Matrices for Testing Associations between Gene Expression Patterns and Related Variables. Proc. Natl. Acad. Sci. USA.

[42] Robinson M.D., McCarthy D.J., Smyth G.K. (2010). edgeR: A Bioconductor Package for Differential Expression Analysis of Digital Gene Expression Data. Bioinformatics.

[43] Boutet E., Lieberherr D., Tognolli M., Schneider M., Bansal P., Bridge A.J., Poux S., Bougueleret L., Xenarios I., Edwards D. (2016). UniProtKB/Swiss-Prot, the Manually Annotated Section of the UniProt KnowledgeBase: How to Use the Entry View. Plant Bioinformatics: Methods and Protocols.

[44] Kanehisa M., Sato Y., Kawashima M., Furumichi M., Tanabe M. (2016). KEGG as a Reference Resource for Gene and Protein Annotation. Nucleic Acids Res..

[45] Buchfink B., Xie C., Huson D.H. (2015). Fast and Sensitive Protein Alignment Using DIAMOND. Nat. Methods.

[46] Chendev Y.G., Sauer T.J., Ramirez G.H., Burras C.L. (2015). History of East European Chernozem Soil Degradation; Protection and Restoration by Tree Windbreaks in the Russian Steppe. Sustainability.

[47] Van Der Heijden M.G., Bardgett R.D., Van Straalen N.M. (2008). The Unseen Majority: Soil Microbes as Drivers of Plant Diversity and Productivity in Terrestrial Ecosystems. Ecol. Lett..

[48] Monciardini P., Cavaletti L., Schumann P., Rohde M., Donadio S. (2003). Conexibacter Woesei Gen. Nov., Sp. Nov., a Novel Representative of a Deep Evolutionary Line of Descent within the Class Actinobacteria. Int. J. Syst. Evol. Microbiol..

[49] Vieira S., Huber K.J., Geppert A., Wolf J., Neumann-Schaal M., Luckner M., Wanner G., Müsken M., Overmann J. (2022). Capillimicrobium Parvum Gen. Nov., Sp. Nov., a Novel Representative of Capillimicrobiaceae Fam. Nov. within the Order Solirubrobacterales, Isolated from a Grassland Soil. Int. J. Syst. Evol. Microbiol..

[50] Chao A., Shen T.-J. (2003). Nonparametric Estimation of Shannon’s Index of Diversity When There Are Unseen Species in Sample. Environ. Ecol. Stat..

[51] Sergaki C., Lagunas B., Lidbury I., Gifford M.L., Schäfer P. (2018). Challenges and Approaches in Microbiome Research: From Fundamental to Applied. Front. Plant Sci..

[52] Skaalsveen K., Ingram J., Clarke L. (2019). The Effect of No-till Farming on the Soil Functions of Water Purification and Retention in North-Western Europe: A Literature Review. Soil Tillage Res..

[53] Degrune F., Theodorakopoulos N., Colinet G., Hiel M.-P., Bodson B., Taminiau B., Daube G., Vandenbol M., Hartmann M. (2017). Temporal Dynamics of Soil Microbial Communities below the Seedbed under Two Contrasting Tillage Regimes. Front. Microbiol..

[54] Deng F., Wang H., Xie H., Bao X., He H., Zhang X., Liang C. (2022). Low-Disturbance Farming Regenerates Healthy Deep Soil toward Sustainable Agriculture—Evidence from Long-Term No-Tillage with Stover Mulching in Mollisols. Sci. Total Environ..

[55] Cai L., Guo Z., Zhang J., Gai Z., Liu J., Meng Q., Liu X. (2021). No Tillage and Residue Mulching Method on Bacterial Community Diversity Regulation in a Black Soil Region of Northeastern China. PLoS ONE.

[56] Six J., Elliott E.T., Paustian K. (2000). Soil Macroaggregate Turnover and Microaggregate Formation: A Mechanism for C Sequestration under No-Tillage Agriculture. Soil Biol. Biochem..

[57] Chueirecc L.M.O., Ferreira M.C., de Andrade D.S., Hungria M., Pedrosa F.O., Hungria M., Yates G., Newton W.E. (2000). Effects of Soil Tillage Management and Crop Rotation on Bradyrhizobia Population. Nitrogen Fixation: From Molecules to Crop Productivity.

[58] Hara S., Morikawa T., Wasai S., Kasahara Y., Koshiba T., Yamazaki K., Fujiwara T., Tokunaga T., Minamisawa K. (2019). Identification of Nitrogen-Fixing *Bradyrhizobium* Associated with Roots of Field-Grown Sorghum by Metagenome and Proteome Analyses. Front. Microbiol..

[59] Agashe R., George J., Pathak A., Fasakin O., Seaman J., Chauhan A. (2024). Shotgun Metagenomics Analysis Indicates *Bradyrhizobium* Spp. as the Predominant Genera for Heavy Metal Resistance and Bioremediation in a Long-Term Heavy Metal-Contaminated Ecosystem. Microbiol. Resour. Announc..

[60] Chaddad Z., Lamrabet M., Bennis M., Kaddouri K., Alami S., Bouhnik O., El Idrissi M.M., Dheeman S., Islam M.T., Egamberdieva D., Siddiqui M.d.N. (2024). Nitrogen-Fixing *Bradyrhizobium* Spp. as Plant Growth-Promoting Bacteria to Improve Soil Quality and Plant Tolerance to Biotic and Abiotic Stresses. Soil Bacteria: Biofertilization and Soil Health.

[61] Jones F.P., Clark I.M., King R., Shaw L.J., Woodward M.J., Hirsch P.R. (2016). Novel European Free-Living, Non-Diazotrophic *Bradyrhizobium* Isolates from Contrasting Soils That Lack Nodulation and Nitrogen Fixation Genes—A Genome Comparison. Sci. Rep..

[62] Li Y., Xiong L., Zeng K., Wei Y., Li H., Ji X. (2023). Microbial-Driven Carbon Fixation in Natural Wetland. J. Basic. Microbiol..

[63] Liu S., Li H., Xie X., Chen Y., Lang M., Chen X. (2024). Long-Term Moderate Fertilization Increases the Complexity of Soil Microbial Community and Promotes Regulation of Phosphorus Cycling Genes to Improve the Availability of Phosphorus in Acid Soil. Appl. Soil Ecol..

[64] Rocabruna P., Domene X., Preece C., Fernández-Martínez M., Maspons J., Penuelas J. (2024). Effect of Climate, Crop, and Management on Soil Phosphatase Activity in Croplands: A Global Investigation and Relationships with Crop Yield. Eur. J. Agron..

[65] Richardson A.E., Barea J.-M., McNeill A.M., Prigent-Combaret C. (2009). Acquisition of Phosphorus and Nitrogen in the Rhizosphere and Plant Growth Promotion by Microorganisms. Plant Soil.

[66] Qin L., Xiao Z., Ming A., Teng J., Zhu H., Qin J., Liang Z. (2024). Soil Phosphorus Cycling Microbial Functional Genes of Monoculture and Mixed Plantations of Native Tree Species in Subtropical China. Front. Microbiol..

[67] Tanuwidjaja I., Vogel C., Pronk G.J., Schöler A., Kublik S., Vestergaard G., Kögel-Knabner I., Mrkonjic Fuka M., Schloter M., Schulz S. (2021). Microbial Key Players Involved in P Turnover Differ in Artificial Soil Mixtures Depending on Clay Mineral Composition. Microb. Ecol..

[68] Liang J.-L., Liu J., Jia P., Yang T., Zeng Q., Zhang S., Liao B., Shu W., Li J. (2020). Novel Phosphate-Solubilizing Bacteria Enhance Soil Phosphorus Cycling Following Ecological Restoration of Land Degraded by Mining. ISME J..

[69] Wang J., Chen Z., Xu C., Elrys A., Shen F., Cheng Y., Chang S. (2021). Organic Amendment Enhanced Microbial Nitrate Immobilization with Negligible Denitrification Nitrogen Loss in an Upland Soil. Environ. Pollut..

[70] Padilla F., Gallardo M., Manzano-Agugliaro F. (2018). Global Trends in Nitrate Leaching Research in the 1960–2017 Period. Sci. Total Environ..

[71] Zhong C., Hu G., Hu C., Xu C., Zhang Z., Ning K. (2024). Comparative Genomics Analysis Reveals Genetic Characteristics and Nitrogen Fixation Profile of *Bradyrhizobium*. iScience.

[72] Suman J., Rakshit A., Ogireddy S.D., Singh S., Gupta C., Chandrakala J. (2022). Microbiome as a Key Player in Sustainable Agriculture and Human Health. Front. Soil Sci..

